# Developing and Comparing 2,6-Anthracene Derivatives: Optical, Electrochemical, Thermal, and Their Use in Organic Thin Film Transistors

**DOI:** 10.3390/ma13081961

**Published:** 2020-04-22

**Authors:** Mikhail Y. Vorona, Nathan J. Yutronkie, Owen A. Melville, Andrew J. Daszczynski, Jeffrey S. Ovens, Jaclyn L. Brusso, Benoît H. Lessard

**Affiliations:** 1 Department of Chemical and Biological Engineering, University of Ottawa, 161 Louis Pasteur, Ottawa, ON K1N 6N5, Canada; mvoro006@uottawa.ca (M.Y.V.); omelv065@uottawa.ca (O.A.M.); 2 Department of Chemistry and Biomolecular Sciences, University of Ottawa, 150 Louis Pasteur, Ottawa, ON K1N 6N5, Canada; nathan.j.yutronkie@gmail.com (N.J.Y.); adasz029@uottawa.ca (A.J.D.); 3 X-Ray Core Facility, University of Ottawa, 150 Louis Pasteur, Ottawa, ON K1N 6N5, Canada; Jeffrey.Ovens@uOttawa.ca

**Keywords:** OTFTs, anthracene, crystal, thin film, transistor, packing, semiconductor, n-type

## Abstract

Anthracene-based semiconductors have attracted great interest due to their molecular planarity, ambient and thermal stability, tunable frontier molecular orbitals and strong intermolecular interactions that can lead to good device field-effect transistor performance. In this study, we report the synthesis of six anthracene derivatives which were di-substituted at the 2,6-positions, their optical, electrochemical and thermal properties, and their single crystal structures. It was found that 2,6-functionalization with various fluorinated phenyl derivatives led to negligible changes in the optical behaviour while influencing the electrochemical properties. Furthermore, the choice of fluorinated phenyl moiety had noticeable effects on melting point and thermal stability (Δ*T*_m_ < 55 °C and Δ*T*_d_ < 65 °C). Bottom-gate top-contact (BGTC) organic thin transistors (OTFTs) were fabricated and characterized using the 2,6-anthracene derivatives as the semiconducting layer. The addition of fluorine groups on the phenyl groups led to a transition from p-type behaviour to n-type behaviour in BGBC OTFTs.

## 1. Introduction

Organic light emitting diodes (OLEDs) [1,2] and other organic electronic devices such as organic photovoltaics (OPVs) [3] and organic thin film transistors (OTFTs) [4] can be fabricated using physical vapour deposition (PVD) at significantly lower temperatures than traditional inorganic semiconductor manufacturing [5,6,7,8]. Therefore, PVD can lead to low-cost, high throughput fabrication of large area electronics. In this capacity, OTFTs demonstrate promise as the cornerstone components of next generation electronic devices [5,7,8,9,10,11,12,13].

The choice of the organic semiconducting (OSC) material is critical to the manufacturability and desired operation of an OTFT Various materials have been examined over the last few decades for use as the OSC in OTFTs [14]. Anthracene, the first organic molecule used to study organic semiconductivity in the late-1950s, and its derivatives are still a promising candidate as an OSC [15,16]. For instance, in 2003 researchers were able to develop anthracene-base molecules with p**-type mobilities as high as 0.02 cm^2^ V^−1^s^−1^ [17,18]. Over the past two decades, hundreds of derivatives have been synthesized, characterized and integrated into devices, providing a sound foundation for the development of structure–property–mobility relationships for anthracene-based OSCs [14,19]. In 2015 Liu et al. reported that 2,6-diphenyl anthracene (2,6-DPA) produced record mobilities [20,21]. There are several factors that account for the performance of anthracene-derivatives; however, the most common are: (1) alignment of the frontier molecular orbital energy levels of the material with the Fermi level of the source and drain electrodes, which corresponds to barrier for electron or hole injection; (2) solid-state molecular packing arrangement in either the herringbone or lamellar motif, along with intermolecular distances between adjacent molecules, which serve a crucial role in charge mobility; and (3) ordered stacking and grain density of the thin film morphology, whereby few boundaries and traps optimize charge mobility [14,22,23]. Environmental stability and operating conditions are also important considerations that affect overall device performance and longevity, but are commonly overlooked when studying OTFT performance. Both p- and n-type OSC devices have been shown to be strongly affected by environmental factors such as temperature, light, humidity exposure and atmosphere (ambient, inert and vacuum) [24,25,26].

X-ray diffraction (XRD) can elucidate the packing structure of a derivative, quantifying the distances between molecules and their supramolecular arrangements in a single crystal. A shorter distance between adjacent molecules results in greater π-orbital overlap, which often leads to greater charge mobility. For example, the tightly packed herringbone motif of 2,6-DPA crystals indicates strong π–π interactions which may contribute to high charge mobility in a less ordered thin-film [20,21,27]. Therefore, the analysis of the crystal structure can provide insight into how anthracene-derivatives pack in a thin film. Chemically modifying an anthracene core by coupling reactions can extend electron delocalization throughout the structure, tune the molecular packing motif, alter the highest occupied molecular orbital (HOMO) and lowest unoccupied molecular orbital (LUMO) energy levels, changing the thermal stability of the derivative. Control of these variables is crucial to obtaining a well-functioning OTFT [14,27,28].

A wide variety of high-mobility 2,6-functionalized anthracene derivatives have been synthesized in the last few years; however, only one 2,6-fluorophenyl anthracene derivative has been reported [14]. In our previous study, we reported a series of 9,10-functionalized anthracene derivatives and fabricated preliminary OTFTs with modest performance [29]. In this study, we report five novel 2,6-fluorophenyl anthracene derivatives and their incorporation into OTFTs. We compare these devices to OTFTs using 2,6-DPA and another 2,6-fluorophenyl anthracene derivative developed in 2004 by Yamashita et al. [30]. We attempt to describe the intermolecular interactions in the single crystals of the derivatives and relate them to OTFT device performance. In doing so, we also characterize their optical, electrochemical and thermal properties to further elucidate structure–property–mobility relationship for anthracene-based semiconductors.

## 2. Materials and Methods

### 2.1. General Methods and Procedures

The reagents 2,6-dibromoanthracene (Lumtec Corp., Taipei, Taiwan), 2,6-diphenylanthracene (Lumtec Corp., Taipei, Taiwan), 4-fluorophenylboronic acid (Oakwood Products Inc., Estill, SC, USA), 3-fluorophenylboronic acid (Oakwood Products Inc., Estill, SC, USA), 2-fluorophenylboronic acid (Oakwood Products Inc., Estill, SC, USA) 3,4,5-trifluorophenylboronic acid (Oakwood Products Inc., Estill, SC, USA) 4-trifluoromethylphenylboronic acid (Oakwood Products Inc., Estill, SC, USA), 3-trifluoromethylphenylboronic acid (Oakwood Products Inc., Estill, SC, USA) potassium carbonate (K_2_CO_3_) (Oakwood Products Inc., Estill, SC, USA), tetrakis(triphenylphosphine)palladium(0) (Pd(PPh_3_)_4_) (Strem Chemicals, Newburyport, MA, USA), toluene, N-methyl-2-pyrrolidone (NMP) (Caledon Laboratories Ltd., Georgetown, ON, Canada), and ethanol were commercially obtained and used as received. All solvents used were ACS grade. Dry nitrogen gas was used as the atmosphere. All reactions were performed under an atmosphere of dry nitrogen.

TGAs were performed in 70 µL alumina crucible using a TGA/DSC 1 Mettler Tolledo instrument (Mettler Tolledo, Columbus, OH, USA) under nitrogen gas with a heating rate of 5.0 °C min^−1^. Mel-Temp apparatus was used to take all melting-points and are reported as uncorrected values. Agilent Technologies Cary 630 FT-IR spectrometer was used to record IR spectra of each compound. A Varian Cary Series 6000 UV-Vis-NIR spectrophotometer (Agilent, Santa Clara, CA, USA) was used to measure the UV-Vis spectra and a Varian Cary Eclipse fluorescence spectrophotometer was used to obtain the photoluminescence spectra. HQGC-grade DCM solutions were used to measure all the UV-Vis and fluorescence spectra in 1 cm precision quartz cuvettes. All NMR spectra were run on the Bruker 400 MHz spectrometer (Bruker, Billerica, MA, USA) in DMSO solution at room temperature. Bruker DektakXT Profilometer (Bruker, Billerica, MA, USA) was used to obtain film thickness measurements. Gas Chromatgraphy/Mass Sectrometry (GC/MS) was performed using Agilent 6890 GC (Agilent, Santa Clara, CA, USA) coupled to Agilent 5975 M equipped with a HP-5MS column (30 m × 250 µm × 0.25 µm), and a flowrate of 1.6 mL min^−1^. The initial oven temperature was 275 °C, held for 15 min, then ramped to 300 °C (40 °C /min) and held for 25 min. A 1,2-dichloroethane and toluene solvent mixture was used for all GC/MS experiments. The same procedure was used for all compounds. Stoichiometric loadings and sublimation temperature varied with each derivative.

#### 2.1.1. Preparation of 2,6-bis(2-fluorophenyl)anthracene (o-FPh)

A bubbled-degassed solution of NMP and water (9:1, 150 mL) was transferred to a mixture of 2,6-dibromoanthracene (1.50 g, 4.46 mmol), 2-fluorophenylboronic acid (1.62 g, 11.60 mmol), K_2_CO_3_ (1.62 g, 11.74 mmol), and Pd(PPh_3_)_4_ (52.6 mg, 0.59 mmol). The reaction was stirred for 16 h at 90 °C. After the reaction was cooled to room temperature, 1.0 M NaOH solution (1.5 L) was added to the reaction. The resulting precipitate was filtered, washed with water, and dried. Sublimation at a temperature range of 185–205 °C under a pressure of 10^−3^ Torr with CO_2_ as a carrier gas, which afforded o-FPh as faint yellow crystals (Yield 1.36 g, 4.04 mmol, 91%). GC/MS reported an elution time of 6.517 min with abundance of 2.2 × 10^5^, and also reported an M^+^ peak of 366.0 m/z compared to a prediction of 366.32 m/z. MP: 195–205 °C. ^1^H NMR (δ, 400 MHz, DMSO): 8.61–8.72 (3H, m), 8.40–8.43 (1H, m), 8.29–8.36 (1H, m), 8.18–8.25 (1H, m), 8.07–8.11 (1H, m), 7.71–7.77 (3H, m), 7.61–7.66 (1H, m), 7.45–7.56 (2H, m), 7.34–7.42 (3H, m). ^19^F NMR (δ, 400 MHz, DMSO): 117.72–117.88 (m). ^13^C NMR (δ, 100 MHz, DMSO): 159.72 (2C), 133.28 (2C), 131.89 (2C), 130.59 (2C), 129.03 (2C), 133.01 (2CH), 131.01 (2CH), 130.21 (2CH), 127.28 (2CH), 125.82 (2CH), 125.51 (2CH), 124.81 (2CH), 114.74 (2CH). FT-IR (ν_max_): 1801 (w), 1705 (w), 1701 (w), 1686 (w), 1664 (w), 1653 (w), 1611 (s), 1575 (w), 1565 (w), 1527 (w), 1495 (s), 1467 (m), 1447 (s), 1402 (m), 1310 (w), 1267 (w), 1262 (w), 1234 (w), 1204 (s), 1156 (w), 1103 (m), 1049 (m), 1017 (w), 941 (w), 904 (s), 872 (m), 840 (w), 820 (m), 796 (s), 749 (s), 712 (m), 710 (w), 667 (m) cm^−1^.

#### 2.1.2. Preparation of 2,6-(3-fluorophenyl)anthracene (m-FPh)

Prepared analogously to o-FPh using 3-fluorophenylboronic acid (1.62 g, 11.60 mmol) yielding an off-white crude solid. Sublimation at a temperature range of 185–205 °C under a pressure of 10^−3^ Torr with CO_2_ as a carrier gas afforded m-FPh as white crystals (Yield 1.31 g, 3.58 mmol, 81%). GC/MS reported an elution time of 6.717 min with abundance of 4.6 × 10^5^, and reported an M^+^ peak of 366.2 m/z compared to a prediction of 366.46 m/z. MP: 250–258 °C. ^1^H NMR (δ, 400 MHz, DMSO): 8.60–8.70 (3H, m), 7.22–7.30 (1H, m). 8.32–8.36 (1H, m), 8.18–8.25 (1H, m), 8.05–8.11 (1H, m),7.89–7.98 (2H, m), 7.74–7.77 (3H, m), 7.52–7.65 (3H, m), 7.34–7.42 (1H, m). ^19^F NMR (δ, 400 MHz, DMSO): 60.80–60.84 (s), 112.60–112.66 (m). ^13^C NMR (δ, 100 MHz, DMSO): 161.02 (2C), 141.77 (2C), 133.34 (2C), 132.19 (2C), 131.29 (2C), 130.18 (2CH), 127.51 (2CH), 127.32 (2CH), 125.79 (2CH), 125.53 (2CH), 122.47 (2CH), 116.33 (2CH), 114.39 (2CH). FT-IR (ν_max_): 2103 (w), 1933 (w), 1803 (w), 1609 (s), 1583 (w), 1521 (m), 1471 (s), 1445 (m), 1394 (m), 1333 (m), 1283 (w), 1238 (w), 1159 (w), 1140 (w), 1120 (w), 1073 (w), 1049 (s), 1014 (m), 964 (m), 913 (m), 900 (s), 865 (m), 840 (m), 796 (s), 740 (w), 710 (s), (m), 667 (w), 665 (w) cm^−1^.

#### 2.1.3. Preparation of 2,6-bis(4-fluorophenyl)anthracene (p-FPh)

Prepared analogously to o-FPh using 4-fluorophenylboronic acid (1.62 g, 11.60 mmol) yielding an off-white crude solid. Sublimation at a temperature range of 185–205 °C under a pressure of 10^−3^ Torr with CO_2_ as a carrier gas afforded p-FPh as white crystals (Yield 1.29 g, 3.57 mmol, 80%). GC/MS reported an elution time of 6.780 min with abundance of 1.1 × 10^6^, and reported an M^+^ peak of 366.2 m/z compared to a prediction of 380.41 m/z. MP: 255–265 °C. ^1^H NMR (δ, 400 MHz, DMSO): 8.59–8.69 (3H, m), 8.37–8.42 (3H, m). 8.16–8.22 (1H, m), 8.05–8.11 (2H, m), 7.85–7.96 (3H, m), 7.66–7.61 (2H, m), 7.33–7.41 (2H, m). ^19^F NMR (δ, 400 MHz, DMSO): 114.86–115.16 (m). ^13^C NMR (δ, 100 MHz, DMSO): 161.77 (2C), 137.18 (2C), 133.45 (2C), 132.28 (2C), 130.68 (2C), 133.22 (2CH), 131.56 (2CH), 131.18 (2CH), 126.79 (2CH), 126.56 (2CH), 125.78 (2CH), 116.02 (2CH), 116.10 (2CH). FT-IR (ν_max_): 1805 (w), 1657 (w), 1606 (m), 1517 (m), 1464 (w), 1444 (m), 1406 (w), 1335 (m), 1301 (w), 1283 (w), 1249 (m), 1178 (w), 1159 (w), 1152 (w), 1100 (w), 1070 (w), 1048 (s), 1012 (m), 962 (m), 913 (m), 900 (s), 865 (m), 845 (m), 796 (s), 733 (w), 710 (s), (m), 691 (w), 654 (w) cm^−1^.

#### 2.1.4. Preparation of 2,6-bis(3-(trifluoromethyl)phenyl)anthracene (m-CF_3_Ph)

Prepared analogously to o-FPh using 3-trifluoromethylbenzeneboronic acid (2.20 g, 11.60 mmol) yielding an off-white crude solid. Sublimation at a temperature range of 195–205 °C under a pressure of 10^−3^ Torr with CO_2_ as a carrier gas afforded m-CF_3_Ph as white crystals (Yield 1.34 g, 2.87 mmol, 64%). GC/MS reported an elution time of 5.613 min with abundance of 4.1 × 10^5^, and also reported an M^+^ peak of 466.3 m/z compared to a prediction of 466.43 m/z. MP: 160–170 °C. ^1^H NMR (δ, 400 MHz, DMSO): 8.61–8.77 (2H, m), 8.52–8.63 (2H, m), 8.17–8.27 (6H, m), 7.91–7.99 (2H, m), 7.77–7.81 (4H, m). ^19^F NMR (δ, 400 MHz, DMSO): 60.90–60.95 (m). ^13^C NMR (δ, 100 MHz, DMSO): 142.2 (2C), 133.32 (2C), 132.19 (2C), 131.52 (2C), 130.58 (2C), 130.74 (2CH), 130.14 (2CH), 129.51 (2CH), 127.37 (2CH), 127.01 (2CH), 126.51 (2CH), 125.02 (2CH), 124.98 (2CH), 124.44 (2C). FT-IR (ν_max_): 1907 (w), 1851 (w), 1797 (w), 1736 (w), 1627 (w), 1529 (w), 1495 (w), 1439 (w), 1411 (w) 1394 (w), 1353 (m), 1327 (s), 1259 (s), 1229 (s), 1173 (m), 1128 (s), 1098 (m), 1072 (s), 1033 (s), 1001 (w), 989 (w), 970 (w), 927 (m), 908 (m), 899 (s), 866 (s), 850 (m), 822 (m), 794 (s), 736 (m), 697 (s), 669 (m), 660 (m) cm^−1^.

#### 2.1.5. Preparation of 2,6-bis(4-(trifluoromethyl)phenyl)anthracene (p-CF_3_Ph)

Prepared analogously to o-FPh using 4-trifluoromethylbenzeneboronic acid (2.20 g, 11.60 mmol) yielding an off-white crude solid. Sublimation at a temperature range of 190–205 °C under a pressure of 10^−3^ Torr with CO_2_ as a carrier gas afforded p-CF_3_Ph as white crystals (Yield 1.22 g, 2.65 mmol, 60%). GC/MS reported an elution time of 6.149 min with abundance of 5.7 × 10^5^, and also reported an M^+^ peak of 466.3 m/z compared to a prediction of 466.43 m/z. MP: 285–290 °C. ^1^H NMR (δ, 400 MHz, DMSO): 8.76–8.83 (1H, m), 8.44–8.57 (2H, m), 8.22–8.29 (1H, m), 8.08–8.14 (2H, m), 7.89–8.00 (3H, m), 7.57–7.75 (7H, m). ^19^F NMR (δ, 400 MHz, DMSO): 60.80–60.85 (m). ^13^C NMR (δ, 100 MHz, DMSO): 145.21 (2C), 132.78 (2C), 131.88 (2C), 131.51 (2C), 129.92 (2C), 129.67 (2CH), 129.43 (2CH), 128.21 (2CH), 127.33 (2CH), 126.57 (2CH), 125.82 (2CH), 125.63 (2CH), 124.39 (2CH), 123.64 (2CH). FT-IR (ν_max_): 1928 (w), 1801 (w), 1737 (w), 1614 (w), 1577 (w), 1542 (w), 1463 (w), 1424 (w), 1408 (w), 1391 (w), 1324 (s), 1281 (w), 1234 (w), 1197 (m), 1178 (m), 1165 (w), 1127 (m), 1111 (m), 1070 (s), 1010 (m), 973 (w), 960(w), 920 (w), 902 (s), 865 (s), 846 (s), 800 (s), 785 (w), 762 (w), 738 (s), 718 (w), 669 (m) cm^−1^.

#### 2.1.6. Preparation of 2,6-bis(3,4,5-trifluorophenyl)anthracene (3,4,5-F_3_Ph)

Prepared analogously to o-FPh using 3,4,5-trifluorophenylbenzeneboronic acid (2.04 g, 11.60 mmol) yielding an off-white crude solid. Sublimation at a temperature range of 190–215 °C under a pressure of 10^−3^ Torr with CO_2_ as a carrier gas afforded 3,4,5-F_3_Ph as white crystals (Yield 1.22 g, 2.79 mmol, 63%). GC/MS reported an elution time of 5.560 min with abundance of 2.6 × 10^6^, and also reported an M+ peak of 438.2 m/z compared to a prediction of 438.37 m/z. MP: 220–230 °C. ^1^H NMR (δ, 400 MHz, DMSO): 8.65–8.70 (1H, m), 8.58–8.63 (1H, m), 8.49–8.53 (1H, m), 8.38–8.41 (1H, m), 8.18–8.27 (2H, m), 8.08–8.13 (1H, m), 7.86–8.00 (4H, m), 7.59–7.66 (1H, m). ^19^F NMR (δ, 400 MHz, DMSO): 60.90–60.93 (s), 134.64–134.78 (d), 162.86–163.22 (m). ^13^C NMR (δ, 100 MHz, DMSO): 151.88 (2C), 151.47 (2C), 137.23 (2C), 135.12 (2C), 132.22 (2C), 133.47 (2C), 130.64 (2C), 128.21 (2CH), 127.39 (2CH), 126.48 (2CH), 124.98 (2CH), 115.02 (2CH), 114.89 (2CH). FT-IR (ν_max_): 1803 (w), 1614 (m), 1592 (w), 1525 (s), 1470 (m), 1439 (m), 1409 (w), 1394 (m), 1362 (m) 1329 (m), 1301 (m), 1277 (w), 1263 (w), 1229 (s), 1173 (m), 1174 (m), 1130 (m), 1102 (w), 1074 (m), 1036 (s), 964 (w), 924 (w), 900 (s), 887 (m), 869 (m), 854 (s), 822 (w), 798 (s), 761 (s), 746 (m), 703 (m), 699 (m), 673 (w), 662 (w) cm^−1^.

### 2.2. Electrochemistry

A glass cell and platinum wires for working, counter and pseudo-reference electrodes was used for cyclic voltammetry in a BASi Epsilon potentiostat. Acetonitrile solutions were used in performing cyclic voltammetry (dried by J. C. Meyer solvent purification system and stored over 3 Å molecular sieves) containing 0.1 M tetrabutylammonium hexafluorophosphate (Oakwood) as supporting electrolyte. A scan rate of 100 mV/s was used for all cyclic voltammetry scans. All experiments were referenced to the Fc/Fc+ redox couple of ferrocene at +0.475 V vs. saturated calomel electrode (SCE).

### 2.3. Thermogravimetric Analysis

70 uL alumina crucible were used for TGA analyses, using a TGA/DSC 1 Mettler Tolledo instrument at a heating rate of 5.0 °C min^−1^ under nitrogen gas. 5% weight loss was used as the decomposition temperature (T_d_) for all the compounds.

### 2.4. Crystallographic Characterization

Crystallographic data collection and processing were performed by the X-Ray Core Facility at the University of Ottawa. Crystals were mounted on MiTeGen sample holders using Parabar oil. Data were collected on a Bruker Smart (o-FPh, m-FPh, and p-FPh) or Kappa (m-CF_3_Ph, p-CF_3_Ph and 3,4,5-F_3_Ph) diffractometer equipped with an ApexII CCD detector and a sealed-tube Mo K source (λ = 0.71073 A). During collection, crystals of m-CF_3_Ph, p-CF_3_Ph and 3,4,5-F_3_Ph were cooled to 213(2) K, crystals of m-FPh and p-FPh were cooled to 200(2) K, and o-FPh was collected at room temperature. Sample cooling was effected via a refrigerated, dry compressed air stream. Raw data collection and processing were performed with the Apex3 software package from Bruker [31]. Initial unit cell parameters were determined from 36 data frames from select ω scans. Semi-empirical absorption corrections based on equivalent reflections were applied [32]. Systematic absences in the diffraction data-set and unit-cell parameters were consistent with the assigned space group. Compound p-CF_3_Ph crystallized as a non-merohedral twin. The twin law was discovered using CELL_NOW, and accounted for in the absorption correction via twinabs. [33] The twin law was also accounted for during final refinements. The initial structural solutions were determined using ShelxT direct methods, [33] and refined with full-matrix least-squares procedures based on F^2^ using ShelXle [34]. Hydrogen atoms were placed geometrically and refined using a riding model. Twin fractions were also refined in ShelXle. The Cambridge Crystallographic Data Centre (CCDC) repository deposition codes for the compounds are as follow: o-FPh (1992977); m-FPh (1992978); p-FPh (1992979); m-CF_3_Ph (1992980); p-CF_3_Ph (1992981); and 3,4,5-F_3_Ph (1992982).

### 2.5. Electrical Characterization

Organic thin film transistors (OTFTs) were fabricated in a bottom gate top contact configuration by PVD onto Si/SiO_2_ organic semiconductor substrates with gold source-drain electrodes deposited atop (*W* = 1000 μm, *L* = 30 μm). Substrate preparation, testing instrumentation and measurement/characterization was performed in accordance with our previous publications [4,35].

## 3. Results and Discussion

### 3.1. Synthesis and Purification of 2,6-Anthracene Derivatives

**Figure 1:**
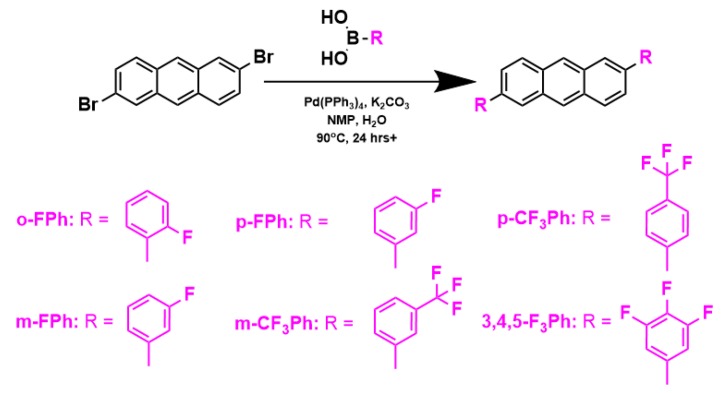
Synthesis of 2,6-disubstituted anthracenes via Suzuki-Miyura cross-coupling reactions using literature methods [35,36].

The palladium-catalyzed Suzuki-Miyura cross-coupling reaction was used to synthesize the 2,6-disubstituted anthracenes (o-FPh, m-FPh, p-FPh, m-CF_3_Ph, p-CF_3_Ph and 3,4,5-F_3_Ph) starting from commercially available reagents, as shown in Figure 1. 2,6-DPA was not synthesized, but rather purchased from a distributor (see Section 2.1). The procedure for the aforementioned cross-coupling reactions was based on literature methods [36,37]. Tetrakis(triphenylphosphine) palladium (0) (Pd(PPh_3_)_4_) was employed as the catalyst in a one-pot reaction with 2,6-dibromoanthracene and varying boronic acids in a degassed solvent mixture of NMP and water. Heating of the mixtures overnight achieved full conversion; completion of the reaction was confirmed by thin layer chromatography. The crude product was isolated by removal of the solvent via vacuum filtration after precipitation in a 1.0 M NaOH solution. The crude was washed with water and dried overnight. Finally, the crude materials were sublimed to obtain each product as a semi-crystalline film in high electronic purity.

### 3.2. Optical and Electrochemical Properties

UV-visible (UV-Vis) and photoluminescence (PL) spectroscopy was conducted on all the compounds in dichloromethane (DCM).The maximum peak absorbance ($\lambda_{\max}^{\mathrm{abs}}$), energy gap ($E_{\mathrm{gap}}$), and photoluminescence maximum peak emissions ($\lambda_{\max}^{\mathrm{em}}$) are reported in Table 1.The respective material spectra can be found in the electronic supplementary information (ESI) (Figures S1–S7). A characteristic triple finger-shape of the compounds is clearly identifiable amongst all the spectra. The characteristic peaks observed between 325–425 nm correspond to the π–π* (S_0_ → S_1_) transitions of the anthracene core [37,38,39,40,41]. The absorption profiles of the compounds display the The $E_{\mathrm{gap}}$ quantities are reported between 2.96–3.04 eV for the compounds which is slightly higher than typically observed for 2,6-disubstituted anthracene derivatives [14,20,27,37,42,43,44,45,46].

The Stokes shifts varied slightly between the derivatives with both of the two trifluoromethylphenyl derivatives, m-CF_3_Ph and p-CF_3_Ph, showing smaller shifts than the fluorophenyl derivatives. Furthermore, there is a lack of mirror image quality and finer details are absent in the emission versus the absorption profiles. This is common, as fluorescent imaging tends to be more sensitive to high background signatures that obscure signals of interest [47]. Lastly, nearly identical emission profiles were observed for the excitation of two higher energy $\lambda_{\max}^{\mathrm{abs}}$, suggesting similar relaxation pathways.

**Figure 2:**
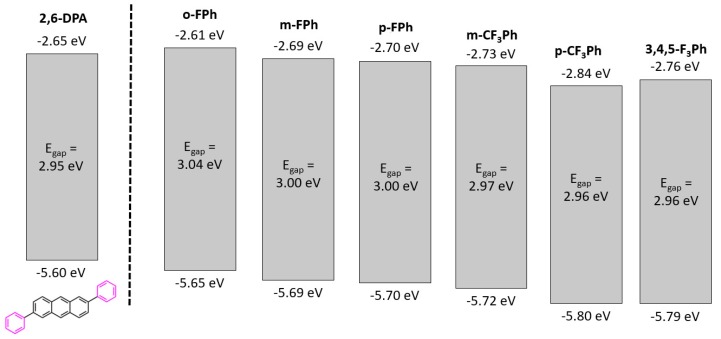
Energy level diagrams of compounds, plus 2,6-DPA for a non-fluorinated baseline [20,21].

The onset of the oxidation potential obtained by cyclic voltammetry (CV) was used to estimate the HOMO level energy. Dilute acetonitrile solutions of each compound were used for CV with 0.1 M *n*-Bu_4_NPF_6_ as the supporting electrolyte [46,48,49,50,51]. The stacked CV spectra can be found in the ESI (Figure S14). By adding the *E*_gap_ from UV-vis to the HOMO energy level determined from CV, the LUMO energy levels could be estimated, resulting in a full energy level diagram for each compound, as shown in Figure 2.

In general, the energy level diagrams suggest there is a noticeable drop in the energy levels of the compounds with more fluorination relative to 2,6-DPA. This is consistent with fluorine-base moieties acting as electron withdrawing groups leading to a drop in the frontier orbital energy levels, especially of the HOMO energy level [52,53,54]. This result indicates that despite the compounds having generally comparable energetics and oxidative stability, there might be a perceptible difference in performance on account of varying charge injection barriers between the Fermi work function of a selected metal electrode and the frontier orbital energy levels.

### 3.3. Thermogravimetric Analysis

**Figure 3:**
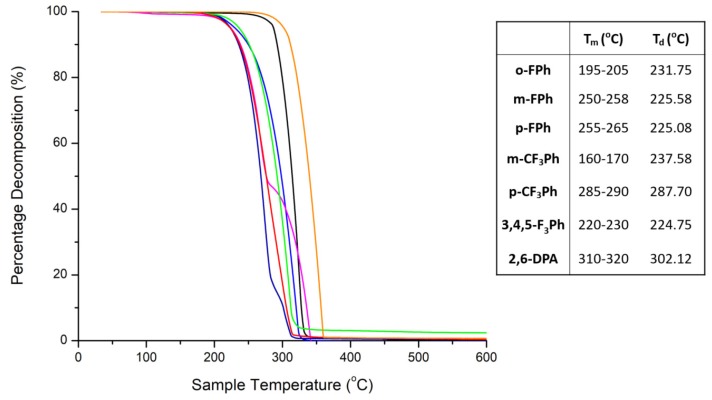
Thermogravimetric analysis curves: o-FPh (blue), m-FPh (magenta), p-FPh (navy), m-CF_3_Ph (green), p-CF_3_Ph (black), 3,4,5-F_3_Ph (red), and 2,6-DPA (orange), as well their associated melting point (*T*_m_) and decomposition temperatures (*T*_d_) (corresponding to 5% weight loss).

The thermal stability of an OSC in a device is equally as important as the energetics for proper device functionality. High phase transition temperatures (i.e., fusion temperature) and decomposition temperatures are optimal in order to avoid morphologically changes in a thin film of a device, which would ultimately lead to its failure. Therefore, the melting points (*T*_m_) and decomposition temperatures (*T*_d_) were measured for each compound and are tabulated in Figure 3. Contrary to the energetic and optical properties, the type of moiety attached to the 2,6-position of the anthracene core resulted in considerable differences in the melting point and decomposition temperature between the molecules. Thermogravimetric analysis (TGA) was used to determine the decomposition temperature whereby 5% weight loss would correspond to the *T*_d_ of a compound. TGA was performed in a nitrogen atmosphere at a ramp heating rate of 5.0 °C min^−1^. 2,6-DPA was examined as well as a non-fluorinated base molecule. It was observed that the decomposition temperature of p-CF_3_Ph was the highest (289 °C) among the compounds that we synthesized and purified–a trifluoromethylphenyl anthracene derivative. The decomposition temperature of the remaining fluorophenyl derivatives were significantly lower (225–232 °C). Interesting, the melting point of p-CF_3_Ph was consistently the highest as well (285–290 °C). These results are indicative of solid-state packing with stronger and more intermolecular interactions, particularly for p-CF_3_Ph and 2,6-DPA that show higher melting point and decomposition temperature.

### 3.4. Single Crystal X-Ray Diffraction

**Figure 4:**
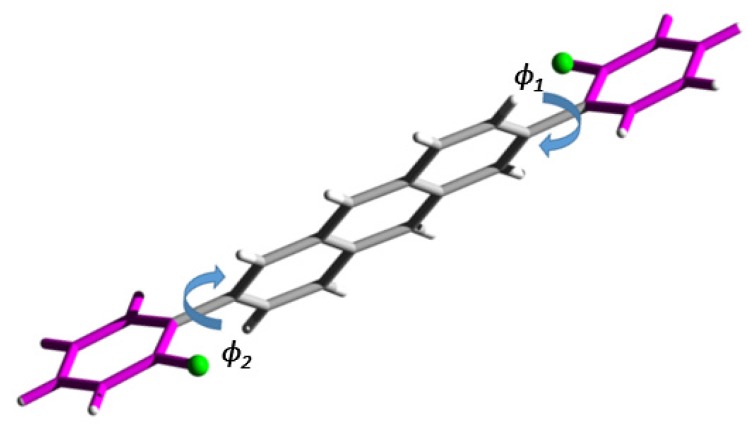
Example of torsion angle location and direction between 2,6-aryl groups to anthracene core of o-FPh derivative (ϕ_1_, ϕ_2_).

**Figure 5:**
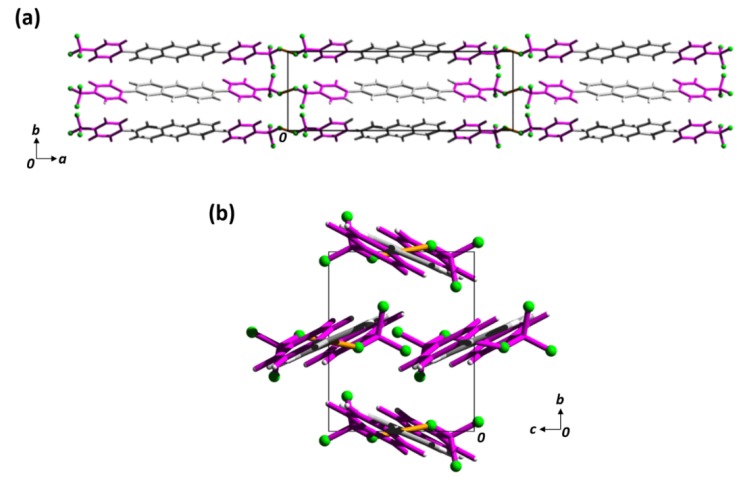
(**a**) Packing of p-CF_3_Ph viewed along the *c*-direction (**b**) and *a*-direction. 2,6-position moieties are shown in magenta, and anthracene cores are shown in grey, while fluorine atoms are shown in green. Short contacts between molecules (within array) are shown in yellow.

To gain insights on the structure–property relationship of 2,6-disubstituted anthracenes, single crystals of the compounds were grown using dynamic vacuum sublimation, and their solid-state architectures were elucidated using X-ray diffraction and are depicted in Figures S8–S13 of the ESI. Crystallographic parameters are presented in Tables S1 and S2 of the ESI. The Cambridge Crystallographic Data Centre (CCDC) repository deposition codes for the compounds’ crystal structures are listed in Section 2.4. At the molecular level, the anthracene frameworks are relatively planar to within 0.028 ± 1.0 × 10^−3^ Å, indicative of little to no distortion about the anthracene core, as observed for other substituted derivatives [29]. Looking at the molecular framework of the seven derivatives, the mean torsion angle φ between the aryl substituents and the anthracene skeleton range between 7.2° and 48.3° (Figure 4). These angles are substantially lower in comparison to their 9,10-substituted isomers, as steric interactions are mitigated with the peri-hydrogen atoms [29]. Amongst the present molecules with monofunctional phenyl groups, an overall trend indicates an increase in ϕ as the fluoro-based group pivots from the para position inwards to the ortho placement (see Table 2). Interestingly, when comparing ϕ between p-FPh and p-CF_3_Ph, the twisting is reduced by almost half. This co-planarization of the aryl groups may be associated with enhanced conjugation of the molecular π-system and the suppression of steric/electronic interactions with the central anthracene.

o-FPh, m-FPh, p-CF_3_Ph, 3,4,5-F_3_Ph crystallize in the monoclinic space group with one half a molecule in the asymmetric unit. p-FPh and m-CF_3_Ph as these derivatives crystallize in the monoclinic space group with one unique and two halves of molecule in the asymmetric unit, respectively. Of the fluorine variants, p-FPh and p-CF_3_Ph adopt a synonymous herringbone arrangement to that of 2,6-DPA, as shown in Figure 5 and Figure S5. The addition of the para-substituent appears to have a subtle influence on the overall crystal packing. For example, the herringbone domains are not as tightly packed in comparison to their phenyl counterpart, which is evident by the diminishing number and elongation of the edge-to-face interactions (i.e., C–H π contacts). In 2,6-DPA, sixteen interactions are present for one molecule within a range of 2.84–2.86 ± 1.0 × 10^−2^ Å, whereas six exist for p-FPh within a slightly larger range of 2.87 − 2.88 ± 1.0 × 10^−2^ Å. As for p-CF_3_Ph, no interactions within the van der Waals separation exist between neighbouring molecules. Additionally, the herringbone angle increases between edge-to-face molecular pairs from p-FPh to p-CF_3_Ph (i.e., DPA = 41.5°; p-FPh = 45.2°; p-CF_3_Ph = 47.9°). Interestingly, this trend is not reflected in the centroid distances between anthracene moieties and suggests the less dense arrangement may not necessarily be a steric response, but rather be attributed to the incremental flattening of the herringbone assembly from the general planarization of the molecular framework.

The impact on the solid-state structure becomes more pronounced as the substituents migrate around the phenyl moiety (i.e., meta and ortho substitution vs. para). For both meta-substituted analogues m-FPh and m-CF_3_Ph, a herringbone assembly can be observed along the short molecular axis of the anthracene core, but deviates from its classical definition of edge-to-face pairs. When viewing m-FPh and m-CF_3_Ph crystals structures along the *a*- and *c*-directions respectively, it becomes apparent that there is an unfavourable degree of slippage along their long molecular axes (Figures S2 and S4). Interestingly, the herringbone angle is only slightly larger than that of its para-substituted cognates (i.e., m-FPh = 49.9°; m-CF_3_Ph = 51.8°) (Table 2) and yet, there is an enhancement in the number and degree of intermolecular interactions between edge-to-face pairs. In spite of these ideal structural traits, the degree of π-stacking s reduced between neighbouring anthracene cores due to the aforementioned slippage. As for the o-FPh, the herringbone motif is replaced with a lamellar-like arrangement of the anthracene units, such that three distinct centroid distances exist between neighbouring molecules (Figure S8). Nonetheless, a herringbone-like arrangement can be seen between adjacent molecules lengthwise (i.e., along the *b*-direction), where edge-to-face interactions occur between the terminal phenyl rings. The dihedral angle between the mean planes of anthracene frameworks is the highest amongst the compounds (i.e., 78.0°), restricting electronic dimensionality in two-dimensions through the lamellar layers parallel to the *ac*-plane.

When 3,4,5-trifluorophenyl substituents are employed along the anthracene backbone, the molecules adopt a herringbone motif resembling that of m-FPh and m-CF_3_Ph (Figure S6). Instead of molecules inclining along the short molecular axis towards each other, the preference lies along the long molecular axis preventing any favourable C–H/F···π or π···π interactions. This is also supported by the lowest herringbone angle of 23.5°. From the various substitution patterns, replacement of ortho and/or meta- hydrogen atoms appear to have a profound effect on the crystallographic packing of 2,6-susbtituted anthracenes.

### 3.5. OTFT Performance

**Figure 6:**
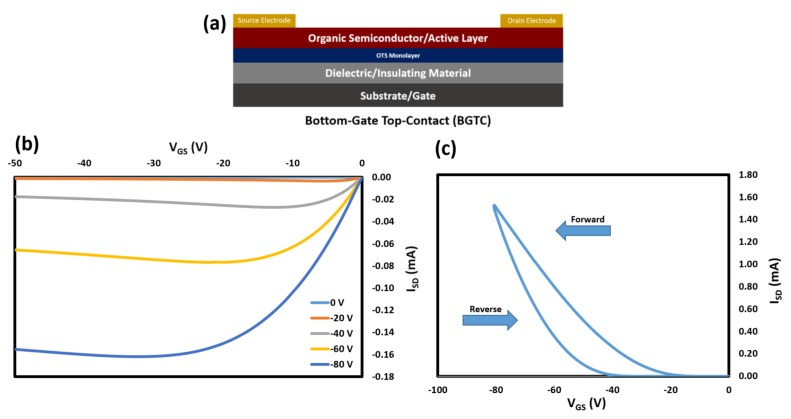
(**a**) OTFT device architecture (bottom-gate top-contact) used to characterize anthracene derivatives. (**b**) Typical output curve of 2,6-DPA fabricated OTFTs. (**c**) Typical transfer curve of 2,6-DPA fabricated OTFTs.

OTFT were fabricated by physical vapour deposition (PVD) of the anthracene semiconductors on Si/SiO_2_ substrates pretreated with octyltrichlorosilane (OTS). A 30 nm thin film of the semiconductor was deposited at a rate of 0.05 Å s^−1^ onto the substrate that was being heated at 50 °C substrates, followed by 50 nm gold electrode in a bottom-gate top-contact configuration (Figure 6a). OTFT characteristics of compounds can be found in Table 3. All OTFTs were characterized at room temperature, first under an inert environment, and followed by second characterization in air. Figure 6 shows typical output and transfer curves of devices using 2,6-DPA as the semiconducting.

All of the compounds, except 3,4,5-F_3_Ph, exhibited either p-type or n-type field-effect mobility. Compounds m-CF_3_Ph and p-CF_3_Ph were the only materials that operated as n-type, but as expected did not produce a field-effect when tested in air, likely due to suppression of n-type behaviour in air [24,25]. The electron mobility, *μ_avg,e_* of p-CF_3_Ph was three orders of magnitude greater than that of m-CF_3_Ph. Overall, the hole mobilities (*μ_avg,h_*) for all of the new compounds were on the order of ≈ 10^−6^ cm^2^ V^−1^ s^−1^ with an average threshold voltage (*V*_T_) between −65 V to −125 V, and *I*_on/off_ ranging between 10^0^–10^2^ (Table 3). As a comparison, 2,6-DPA was purchased and purified by train sublimation prior to integration into devices. Liu et al. reported the molecular packing and intermolecular interactions of 2,6-DPA are ideally suited for favourable device performance, whereby 2,6-DPA stacks in an edge-on upright orientation, ensuring optimal hole conduction occurs parallel to the channel. This, in combination with relatively short π–π contacts between neighboring molecules (δ_plane_) and strong C–H···π interactions, contribute to exceptionally high *μ_avg,h_*. The use of both 2,6-DPA and p-CF_3_Ph have previously been reported in the literature with values similar to our findings [20,21,30]. Overall, the functionalization of the 2,6-positions is proving to have a much more significant effect on device performance compared to functionalization of the 9,10 positions [29].

The OTFT performance of o-FPh, m-FPh, p-FPh and m-CF_3_Ph were modest, most likely due to weak intermolecular interactions and unfavourable molecular packing in thin film as discussed in the x-ray diffraction section, despite having comparable π–π contact distances (δ_plane_) to 2,6-DPA, as observed by X-ray crystallography (Table 3). Specifically, the herringbone domains of these molecules are not as tightly packed in comparison to 2,6-DPA, which is evident by the diminishing number and elongation of the edge-to-face interactions (i.e., C–H···π contacts). Ultimately, this leads to detrimental effects on π-stacking, which in turn would cause poor electrical charge conduction. This is corroborated by the relatively low melting point and decomposition temperatures of the materials as found in the thermogravimetric analysis section, in comparison to the higher performing 2,6-DPA and p-CF_3_Ph, suggesting a correlation of lower performing materials to much weaker intermolecular interactions. p-FPh performed poorly even though its molecular packing is analogous to 2,6-DPA and p-CF_3_Ph; however, its melting point and decomposition temperature are similar to the rest of the low performing materials.

While the p-type performance of o-FPh, m-FPh, p-FPh, m-CF_3_Ph, and 3,4,5-F_3_Ph were modest due to weak intermolecular interactions and unfavourable molecular packing, it is still likely that the *μ_avg,h_*, and the *V*_T_ of 2,6-DPA can be improved with interlayer engineering. 2,6-DPA is an excellent candidate for such device optimization as the morphological and thin-film properties are ideal under non-engineered conditions. Careful application of interlayer materials such as MoO_3_ and N,N_0_-bis(3-methylphenyl)-N,N_0_-diphenylbenzidine (TPD) beneath the Au electrode contacts of a BGTC device can have the effect of reducing the injection barrier by sculpting the charge injection profile relative to the Fermi level of Au and the HOMO of the OSC [55,56]. Analogous contact engineering techniques, such as the use of Mn or Cr interlayers, may be applied to n-type compounds m-CF_3_Ph and p-CF_3_Ph [57].

When further examining the electrochemical characterization, it becomes evident that fluorination of the molecules lowers both the LUMO and the HOMO energy levels, which favours electron transport. Both m-CF_3_Ph and p-CF_3_Ph experienced the greatest drop in HOMO/LUMO levels and they exhibited an *μ_avg,e_*. 3,4,5-F_3_Ph also experienced a significant drop of their HOMO/LUMO energy levels, however, did not exhibit any field-effect likely associated with its large π–π contact distances (δ_plane_) (3.602 Å) relative to all other derivatives. This is corroborated by X-ray diffraction that depict loosely arranged herringbone domains diminishing edge-to-face interactions (i.e., C–H···π contacts) of these molecules. The transition from p-type to n-type upon the addition of peripheral fluorine atoms has previously been reported for several materials such as copper phthalocyanines [12,49,58] or pentacene [59]. These results suggest that functionalization of the 2,6-positions are powerful handles which can be used to modulate the hole and electron mobility. Furthermore, 2,6-position functionalization simultaneously has significant effects on molecular packing and intermolecular interactions which must be taken into account as a way to improve OTFT performance.

## 4. Conclusions

Six novel 2,6-anthracene-based molecules were synthesized and their optical, electrochemical, thermal properties and single crystal structures were characterized. It was found that functionalization of the 2,6-positions with various fluorinated phenyl derivatives results in negligible changes in optical behaviour, while dropping the frontier orbital energy levels, especially the HOMO. Moreover, the choice of fluorinated phenyl moiety had noticeable effects on the melting point and thermal stability (Δ*T*_m_ < 55 °C and Δ*T*_d_ < 65 °C). Organic thin transistors (OTFTs) were fabricated and characterized using the compounds as the semiconducting layer. With the addition of fluorine groups we observed an overall transition from p-type behaviour to n-type behaviour with Au contacts. We also found that the choice of substituent in the 2,6 position led to significant changes to the solid-state arrangement and device performance. These results indicate that functionalization of the 2,6-position of an anthracene core can offer a straightforward way to develop new n-type semiconductors, as well as a powerful handle to potentially improve OTFT device performance.

## Figures and Tables

**Table 1:** Electrochemical and optical properties of compounds.

	$E_{1/2}$ (V) ^a^	$E_{\mathrm{HOMO}}$ (eV) ^b^	$\lambda_{\max}^{\mathrm{abs}}$ (nm)	$E_{\mathrm{gap}}$ (eV) ^c^	$\lambda_{\max}^{\mathrm{em}}$ (nm)	Stokes Shift (nm)
2,6-DPA ^d^	–	−5.60	360, 379, 400	2.95	411, 436	33
o-FPh	0.86	−5.69	351, 368, 388	3.00	407, 431	40
m-FPh	0.89	−5.69	365, 380, 398	3.04	411, 436	46
p-FPh	0.91	−5.70	345, 366, 387	3.00	412, 436	46
m-CF_3_Ph	0.92	−5.72	359, 377, 399	2.97	412, 436	35
p-CF_3_Ph	0.99	−5.80	361, 380, 401	2.96	412, 441	36
3,4,5-F_3_Ph	0.99	−5.79	357, 369, 389	2.96	413, 436	44

^a^. Voltage versus saturated calomel electrode (SCE). ^b^. $E_{HOMO}=-4.80\;eV-\left(E_{onset}^{ox}\;vs\times \frac{Fc}{Fc^{+}}\right).$^c^. The lowest energy absorbance peak from onset was used to calculate the $E_{gap}$; ^d^. $E_{HOMO}$ obtained from Liu et al. [20].

**Table 2:** Torsion angle between 2,6-aryl groups to anthracene core (ϕ), herringbone angles, centroid distances and mean plane separation of π-molecule.

	*ϕ*_1_, *ϕ*_2_ (°)	Herringbone Angle (°)	Centroid Distances (Å)	Plane Distances (Å)
2,6-DPA ^a^	20.6, 20.6	41.5	6.24	2.21
o-FPh	48.3, 48.3	78.0	6.37, 7.00, 7.40	2.43, 4.24, 1.81
m-FPh ^b^	24.0, 24.0 27.3, 27.3	49.9	5.91	2.48, 2.50
p-FPh ^c^	13.2, 13.2 14.7, 14.7	45.2	6.07	2.33
m-CF_3_Ph	26.2, 26.2	51.8	6.14	2.68
p-CF_3_Ph	7.2, 7.2	47.9	6.13	2.48
3,4,5-F_3_Ph	39.3, 39.3	23.5	6.99	6.77

Error of the angle measurements is ± 1.0 × 10^−1^ °. Error of the distances measurements is ± 1.0 × 10^−2^ Å; ^a^. Values obtained Liu et al. XRD crystal structure [20]. ^b^. m-FPh contains two unique asymmetric units; ^b^. Two unique halves of a molecule in the asymmetric unit; ^c^. One unique molecule in the asymmetric unit.

**Table 3:** Summary of organic thin film transistor performance of 2,6-anthracene semiconductors ^a^.

Compound	*δ_plane_*^b^ (Å)	Testing Atmosphere	*μ_avg,h_* (p-type) (cm^2^ V^−1^ s^−1^)	*μ_avg,e_* (n-type) (cm^2^ V^−1^ s^−1^)	*V_T, avg_* (V)	n	*I_on/off_*
2,6-DPA	2.850	N_2_	2.71 ± 1.04	–	−51.0 ± 4.9	57	10^7^
		Air	0.145 ± 0.079	–	−49.9 ± 10.8	58	10^6^
o-FPh	2.430, 4.344, 1.813	N_2_	7.43 ± 2.78 × 10^−5^	–	−64.8 ± 4.0	32	10^1^
		Air	2.34 ± 1.42 × 10^−5^	–	−87.0 ± 12.3	28	10^1^
m-FPh	2.486, 2.503	N_2_	1.74 ± 2.02 × 10^−6^	–	−65.9 ± 22.7	28	10^1^
		Air	8.08 ± 3.45 × 10^−8^	–	−122.2 ± 11.3	35	10^1^
p-FPh	2.330	N_2_	5.89 ± 0.41 × 10^−6^	–	−87.3 ± 30.4	31	10^2^
		Air	3.52 ± 6.96 × 10^−5^	–	−75.0 ± 25.9	23	10^1^
m-CF_3_Ph	2.549	N_2_	–	5.48 ± 3.48 × 10^−6^	79.8 ± 21.2	20	10^1^
		Air	–	–	–	–	–
p-CF_3_Ph	2.482	N_2_	–	3.11 ± 1.27 × 10^−3^	54.7 ± 1.1	35	10^3^
		Air	–	–	–	–	–
3,4,5-F_3_Ph	3.602	N_2_	–	–	–	–	–
		Air	–	–	–	–	–

^a^. OTFTs were characterized to gate voltages of −80 V; Channel length = 30 μm and electrode width = 3000 μm, where I_on/off_ are orders of magnitude of on/off current ratios, μ_avg_ = average mobility, V_T, Avg_ = average threshold voltage, and V_T,max_ = average threshold voltage; ^b^. π–π contacts between adjacent molecules obtained by use of single crystal X-ray diffraction.

## Supplementary Materials

The following are available online at https://www.mdpi.com/1996-1944/13/8/1961/s1, Figures S1–S7: UV-Vis absorption spectrum and emission spectra normalized for comparison for all compounds Figures S8–S13: Single crystal X-ray diffraction views and interactions. Figure S14: Cyclic voltammetry. Table S1. Crystallographic parameters for all compounds. Table S2. Distances (Å) between the individual carbon atoms and the mean plane of the anthracene moiety.
