# Supplementary material for: Developing and Comparing 2,6-Anthracene Derivatives: Optical, Electrochemical, Thermal, and Their Use in Organic Thin Film Transistors
Source: Materials (Basel). 2020 Apr 22;13(8):1961. doi: 10.3390/ma13081961 (PMC7215602; doi:10.3390/ma13081961)
Supplement: Supplementary file 1 [file materials-13-01961-s001.pdf]

materials

Supplementary Materials

Developing and Comparing 2,6-anthracene Derivatives: Optical, Electrochemical, Thermal, and Their Use in Organic Thin Film Transistors

Mikhail Y. Vorona [1], Nathan J. Yutronkie [2], Owen A. Melville [1], Andrew J. Daszczynski [2], Jeffrey S. Ovens [3], Jaclyn L. Brusso [2,*] and Benoît H. Lessard [1,*]

[1] Department of Chemical and Biological Engineering, University of Ottawa, 161 Louis Pasteur, Ottawa K1N 6N5, ON, Canada; mvoro006@uottawa.ca (M.Y.V.); omelv065@uottawa.ca (O.A.M.)

[2] Department of Chemistry and Biomolecular Sciences, University of Ottawa, 150 Louis Pasteur, Ottawa K1N 6N5, ON, Canada; nathan.j.yutronkie@gmail.com (N.J.Y.); adasz029@uottawa.ca (A.J.D.)

[3] X-Ray Core Facility, University of Ottawa, 150 Louis Pasteur, Ottawa K1N 6N5, ON, Canada; Jeffrey.Ovens@uOttawa.ca

* Correspondence: benoit.lessard@uottawa.ca (B.H.L.); jbrusso@uottawa.ca (J.L.B.)

Received: 27 March 2020; Accepted: 15 April 2020; Published: date

Table S1. Crystallographic parameters for all compounds.

	**o-FPh**	**m-FPh**	**p-FPh**	**m-CF₃Ph**	**p-CF₃Ph**	**3,4,5-F₃Ph**
Empirical Formula	$C_{26}H_{16}F_2$	$C_{26}H_{16}F_2$	$C_{26}H_{16}F_2$	$C_{28}H_{16}F_6$	$C_{28}H_{16}F_6$	$C_{26}H_{12}F_6$
Formula Weight, g/mol	366.39	366.39	366.39	466.41	466.41	438.36
Crystal System	monoclinic	monoclinic	monoclinic	monoclinic	monoclinic	monoclinic
Space Group	$P\ 2_1/n$	$P\ 2_1/c$	$P\ 2_1/n$	$P\ 2_1/c$	$P\ 2_1/c$	$P\ 2_1/c$
a, Å	6.2709(14)	10.8415(10)	6.0700(6)	17.5128(18)	21.503(7)	10.8934(6)
b, Å	21.658(4)	5.9169(5)	7.4644(7)	5.8410(5)	7.538(2)	6.9223(4)
c, Å	7.0032(3)	27.714(3)	38.075(4)	11.0028(9)	6.1331(16)	13.2633(8)
α, °	90	90	90	90	90	90
β, °	112.499(6)	94.929(3)	92.381(3)	108.187(5)	94.24(2)	113.297(3)
γ, °	90	90	90	90	90	90
V, Å^3	878.746(10)	1771.23(3)	1723.65(3)	1069.27(17)	991.391(2)	918.606(9)
Z	2	4	4	2	2	2
T, K	298 (2)	200 (2)	200 (2)	213 (2)	213 (2)	213 (2)
ρ_{calc}, g/cm^3	1.385	1.374	1.412	1.449	1.563	1.585
μ, mm^{-1}	0.954	0.093	0.096	0.120	0.129	0.134
$2\theta_{max}$, °	28.297	28.367	26.660	33.357	25.509	25.367
Total/Unique Reflections	2184/1760	4421/2703	3588/1572	3978/2232	1904/1228	1686/1049
Reflections $[I_o \geq 2\sigma(I_o)]$	16697	33190	21086	12978	1904	8786
Parameters/Restraints	127/0	253/0	253/0	185/73	183/102	145/0
R_1, wR_2 $[I_o \geq 2\sigma(I_o)]^a$	0.0496, 0.1291	0.0533, 0.1360	0.0604, 0.1505	0.0714, 0.2023	0.0701, 0.1913	0.0482, 0.1178
Goodness of Fit	1.071	1.031	0.990	1.060	1.063	1.015

[a] Function minimized: $\sum w(F_o^2 - F_c^2)^2$. $R_1 = \sum \left||F_o| - |F_c|\right| / \sum |F_o|$ and $wR_2 = [\sum (F_o^2 - F_c^2)^2 / \sum F_o^4]^{\frac{1}{2}}$

Table S2. Distances (Å) between the individual carbon atoms and the mean plane of the anthracene moiety.

	**C1 (C15)**	**C2 (C16)**	**C3 (C17)**	**C4 (C18)**	**C5 (C19)**	**C6 (C20)**	**C7 (C21)**	**C8 (C22)**	**C9 (C23)**	**C10 (C24)**	**C11 (C25)**	**C12 (C26)**	**C13 (C27)**	**C14**
o-FPh	0.979	1.091	0.235	0.704	0.786	0.057	0.010	0.004	0.016	0.011	0.011	0.003	0.001	–
*m-FPh	0.676	0.738	0.252	0.336	0.420	0.090	0.022	0.001	0.023	0.018	0.009	0.015	0.000	0.367
	(0.290)	(0.208)	(0.657)	(0.602)	(0.085)	(0.024)	(0.000)	(0.023)	(0.022)	(0.007)	(0.016)	(0.004)	–	–
p-FPh	0.017	0.040	0.028	0.009	0.033	0.002	0.040	0.217	0.628	0.833	0.627	0.226	0.023	0.013
	(0.037)	(0.019)	(0.019)	(0.023)	(0.004)	(0.051)	(0.196)	(0.567)	(0.711)	(0.509)	(0.168)	(0.006)	–	–
m-CF_3Ph	0.002	0.022	0.019	0.005	0.012	0.005	0.020	0.088	0.655	0.718	0.238	0.304	0.939	0.388
p-CF_3Ph	0.008	0.025	0.022	0.017	0.036	0.019	0.041	0.186	0.101	0.246	0.480	0.635	0.563	0.423
3,4,5-F_3Ph	0.013	0.000	0.008	0.016	0.006	0.008	0.003	0.005	0.774	0.768	0.025	0.737	0.754	–

* Compound m-FPh has two distinct asymmetric units.

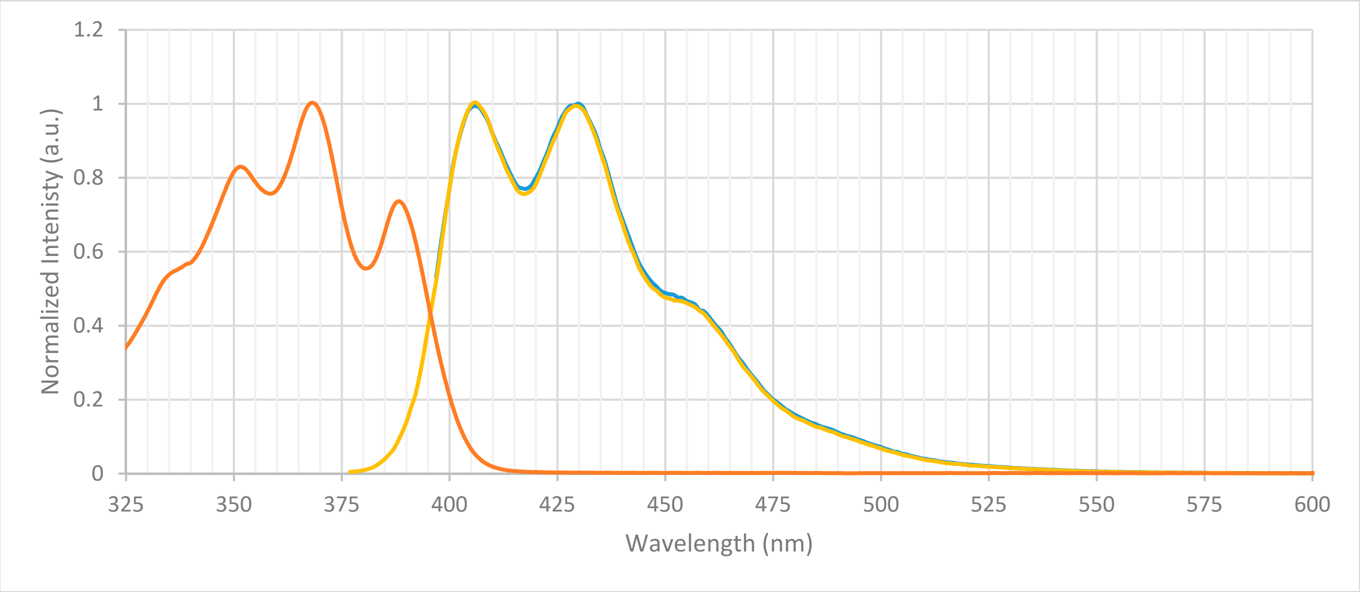

Figure S1. UV-Vis absorption (orange) spectrum and emission spectra with excitation at 388 nm (blue), 375 nm (yellow) and 396 nm (teal) of a DCM solution of o-FPh. Absorption and emission spectra has been normalized for comparison.

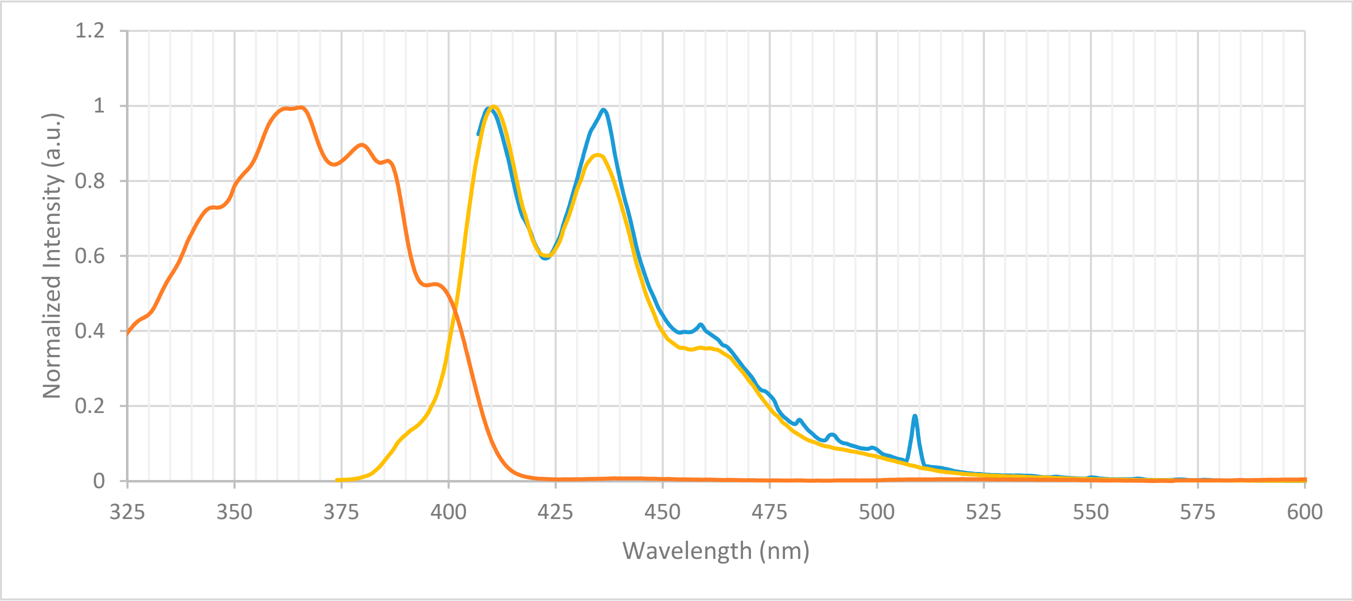

Figure S2. UV-Vis absorption (orange) spectrum and emission spectra with excitation at 398 nm (blue) and 365 nm (yellow) of a DCM solution of m-FPh. Absorption and emission spectra has been normalized for comparison.

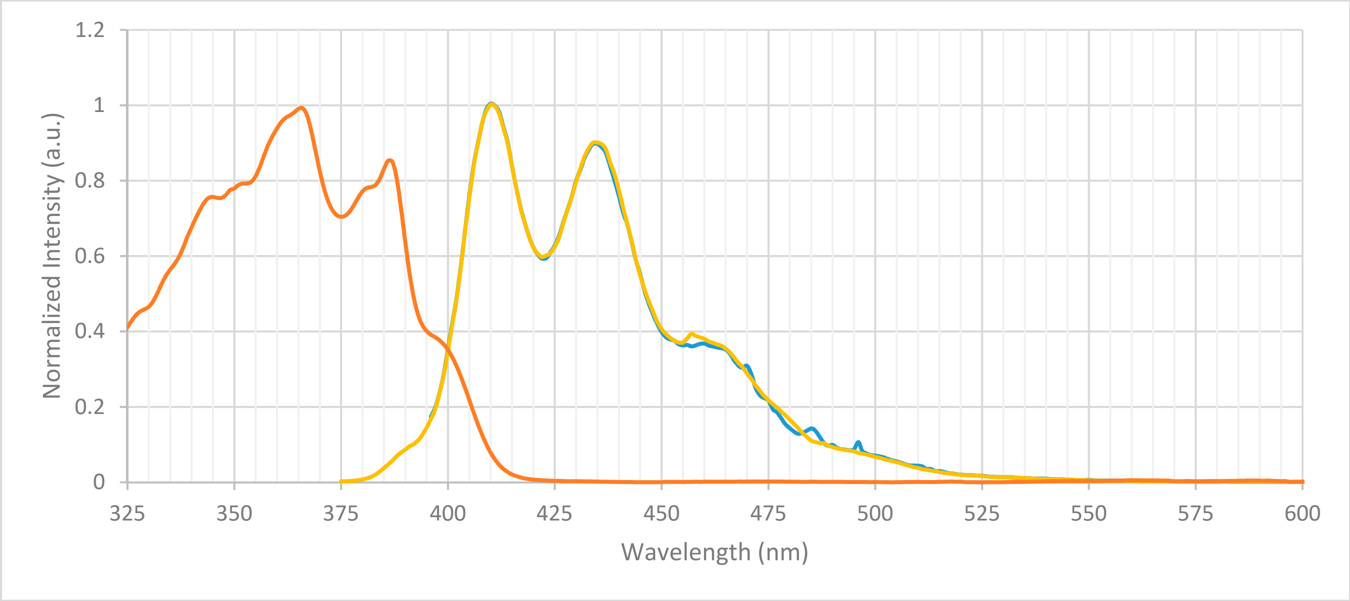

Figure S3. UV-Vis absorption (orange) spectrum and emission spectra with excitation at 396 nm (blue) and 366 nm (yellow) of a DCM solution of p-FPh. Absorption and emission spectra has been normalized for comparison.

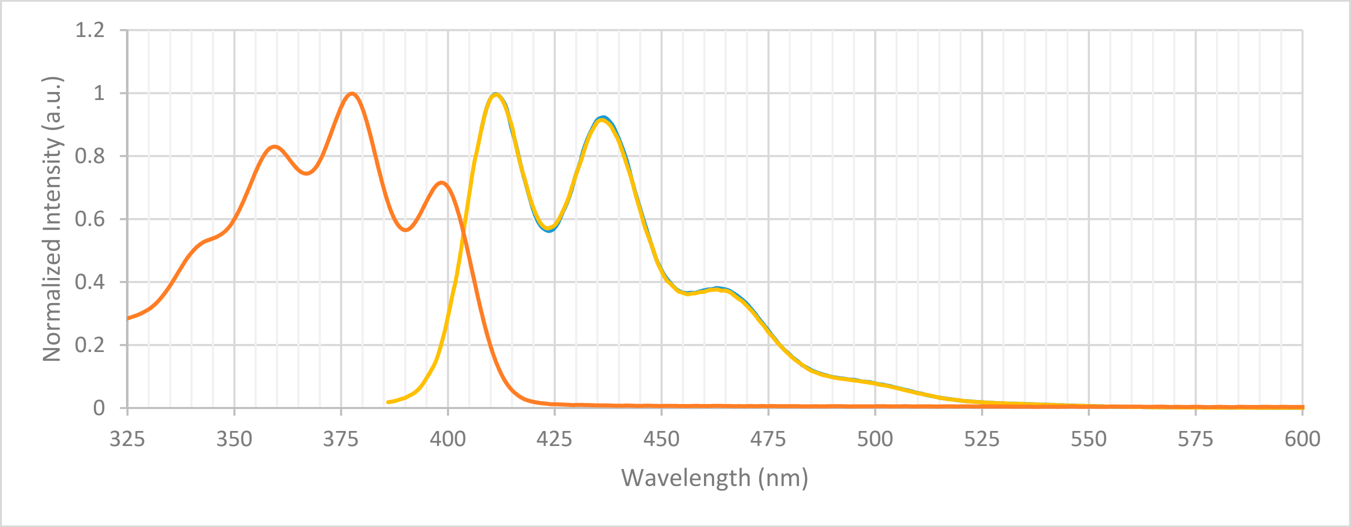

Figure 4. UV-Vis absorption (orange) spectrum and emission spectra with excitation at 357 nm (blue) and 376 nm (yellow) of a DCM solution of m-CF_3Ph. Absorption and emission spectra has been normalized for comparison.

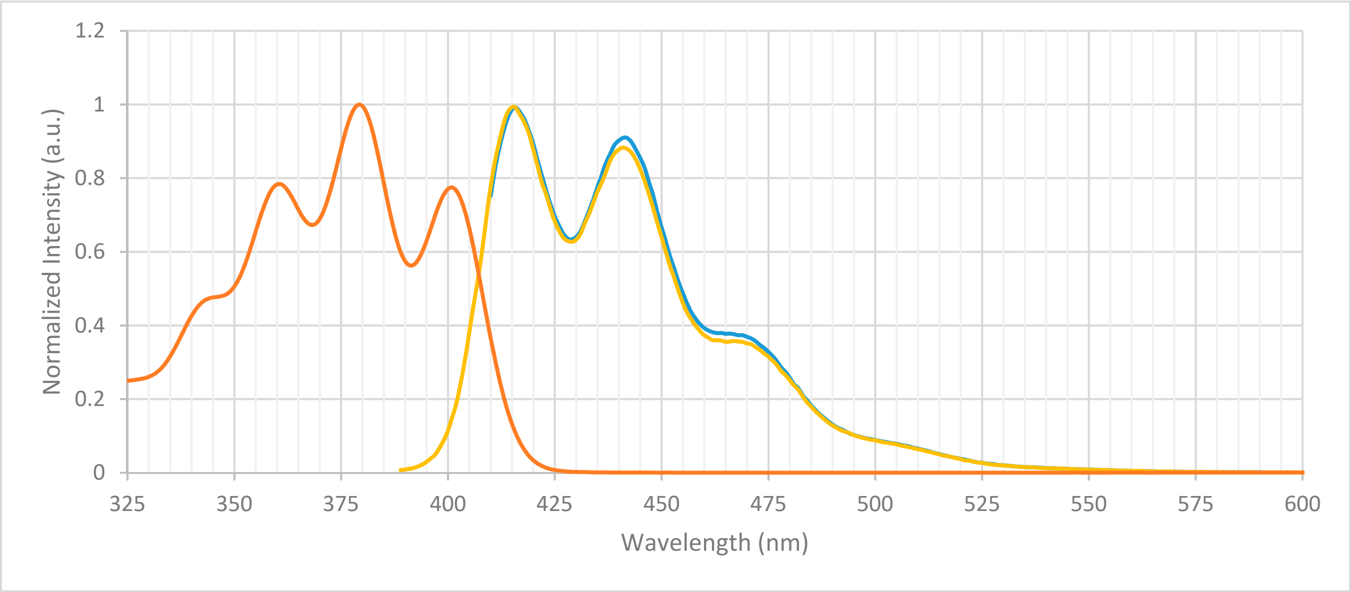

Figure S5. UV-Vis absorption (orange) spectrum and emission spectra with excitation at 401 nm (blue) and 380 nm (yellow) of a DCM solution of p-CF_3Ph. Absorption and emission spectra has been normalized for comparison.

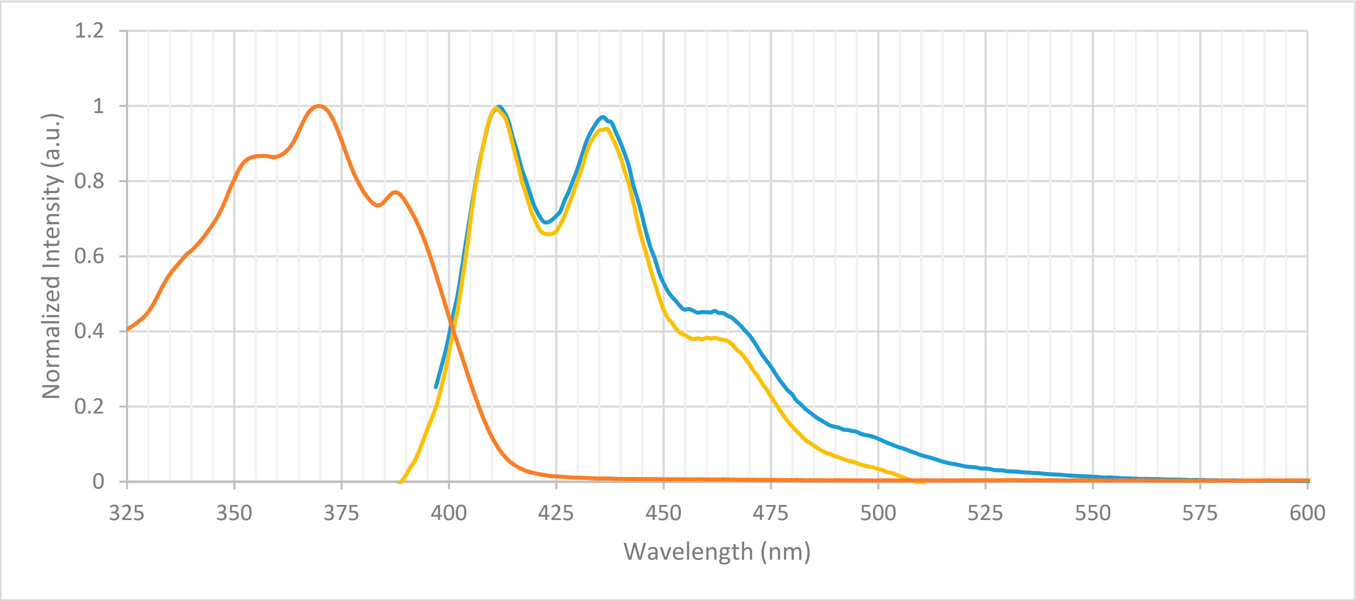

Figure S6. UV-Vis absorption (orange) spectrum and emission spectra with excitation at 389 nm (blue) and 369 nm (yellow) of a DCM solution of 3,4,5-F_3Ph. Absorption and emission spectra has been normalized for comparison.

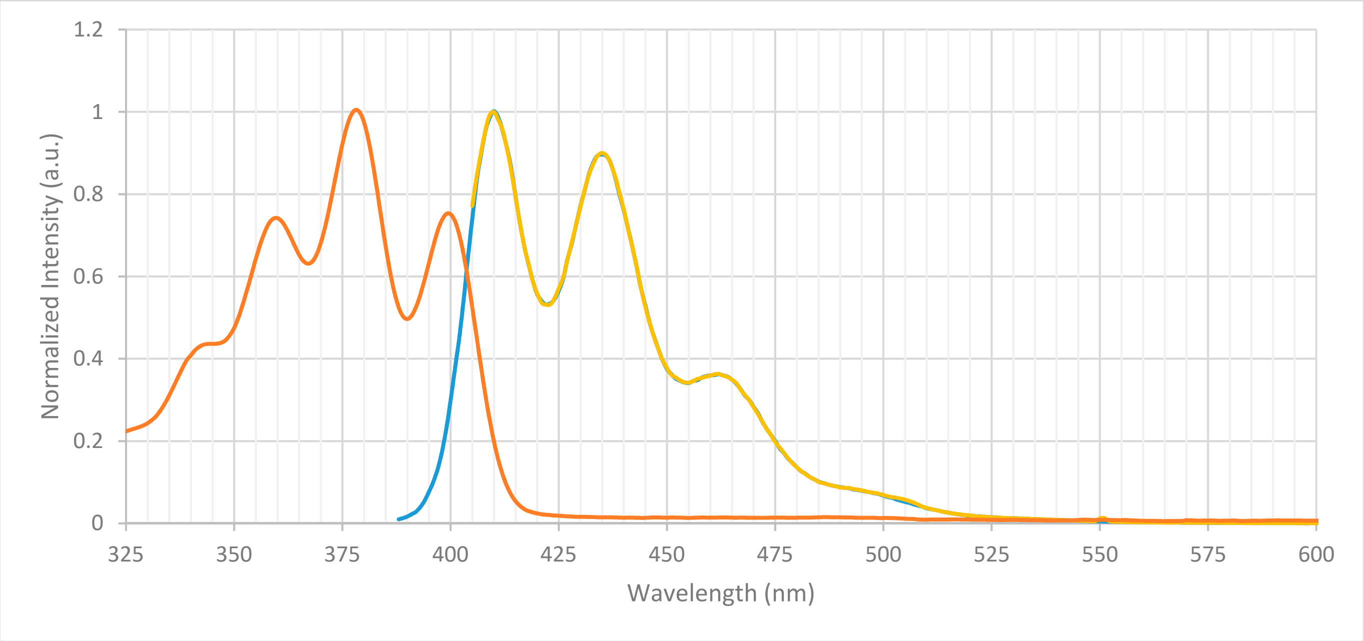

Figure S7. UV-Vis absorption (orange) spectrum and emission spectra with excitation at 379 nm (blue) and 400 nm (yellow) of a DCM solution of 2,6-DPA. Absorption and emission spectra has been normalized for comparison.

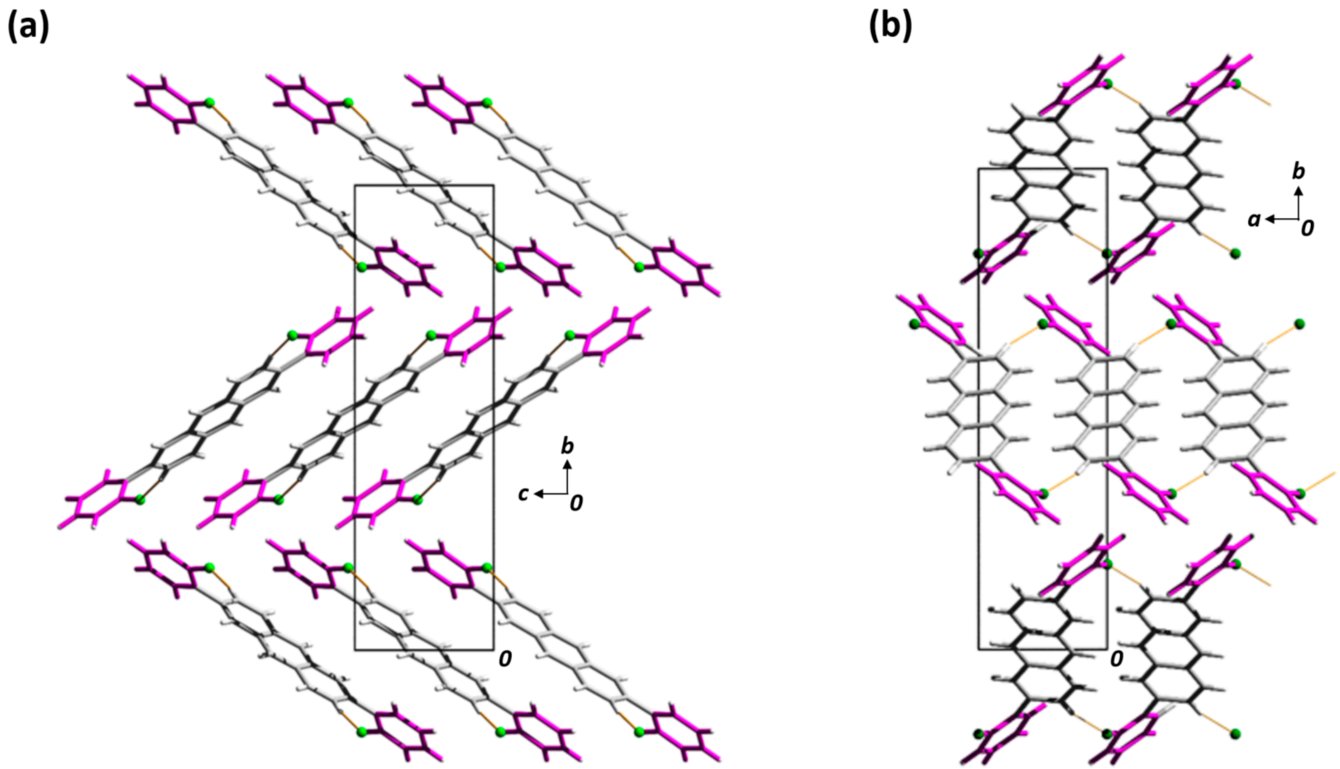

Figure S8. (**a**) Molecular packing of o-FPh viewed down *a*-direction (**b**) and *c*-direction. 2,6-position moieties are shown in magenta, and anthracene cores are shown in grey, while fluorine atoms are shown in green. Short contacts between molecules (within array) are shown in yellow.

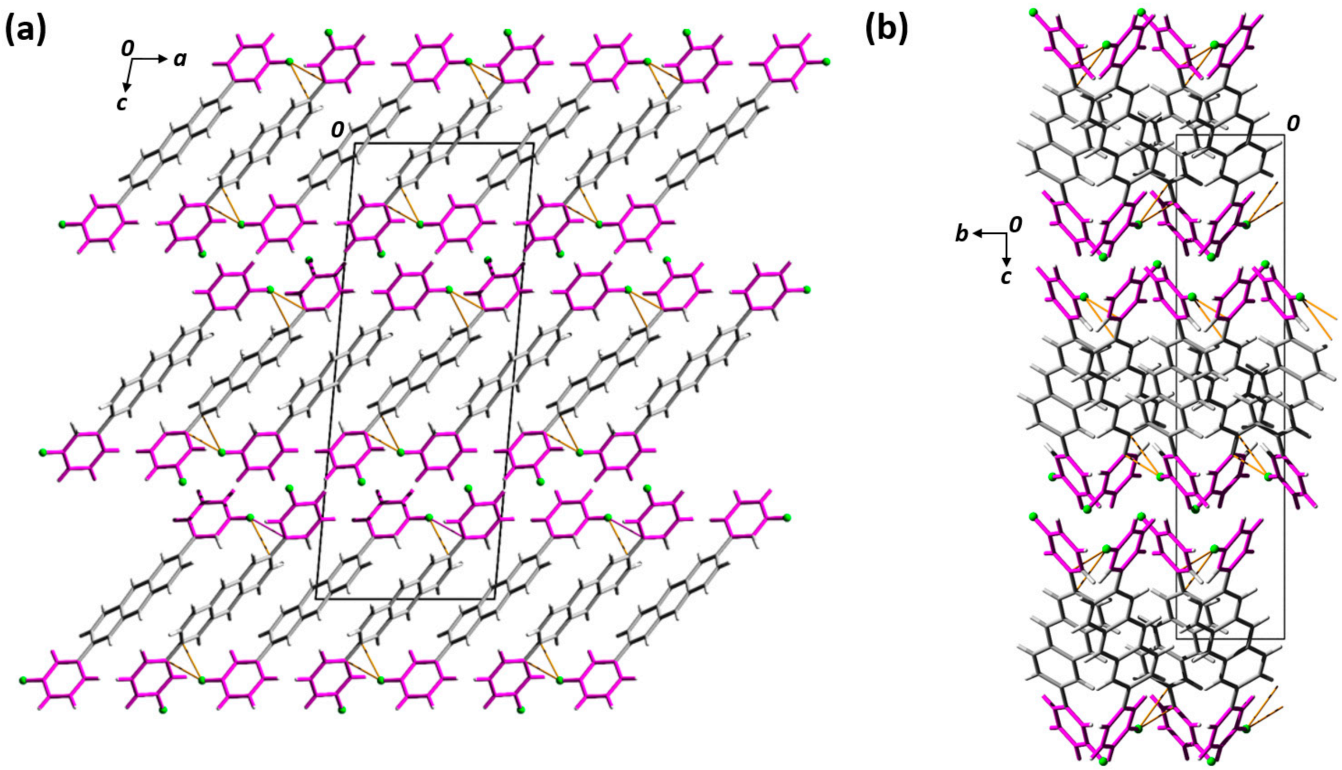

Figure S9. (**a**) Packing motif of m-FPh along the *b*-direction. (**b**) Interaction of two intercalating unit cells as viewed down the a-direction. 2,6-position moieties are shown in magenta, and anthracene cores are shown in grey, while fluorine atoms are shown in green. Short contacts between molecules (within array) are shown in yellow.

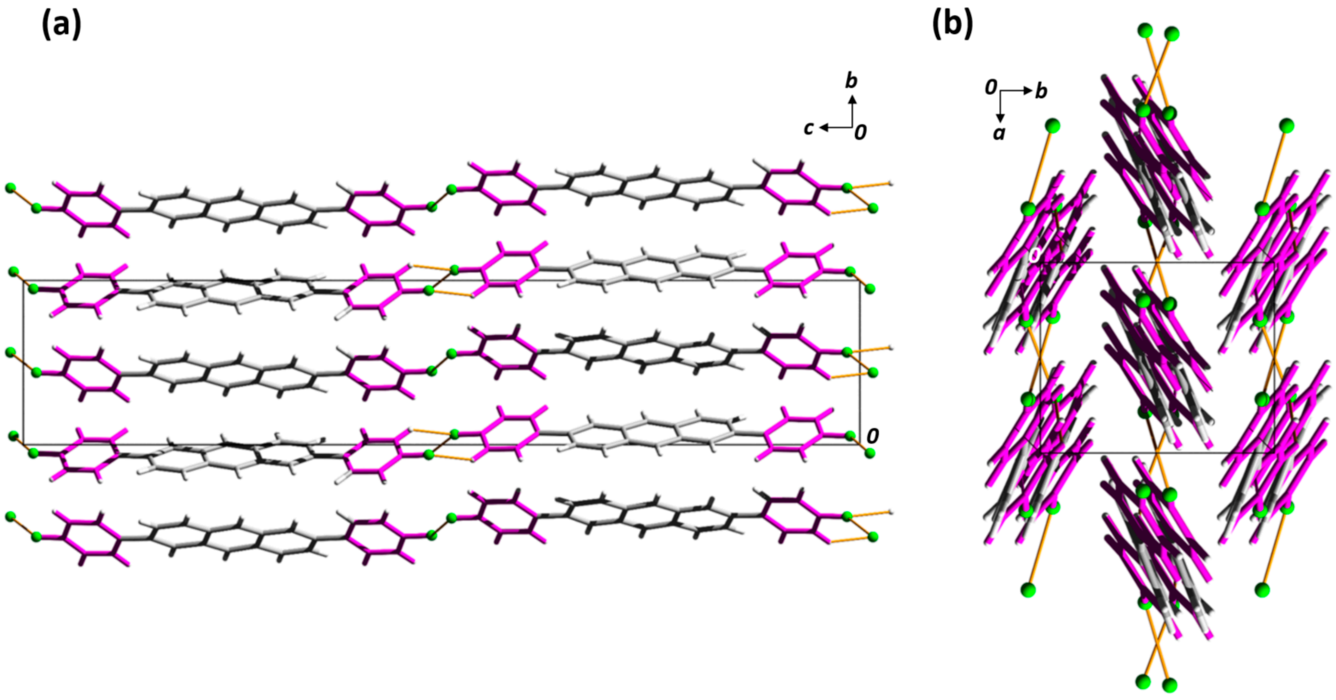

Figure S10. (**a**) Packing motif of p-FPh viewed along the *a*-direction (**b**) and *c*-direction. 2,6-position moieties are shown in magenta, and anthracene cores are shown in grey, while fluorine atoms are shown in green. Short contacts between molecules (within array) are shown in yellow.

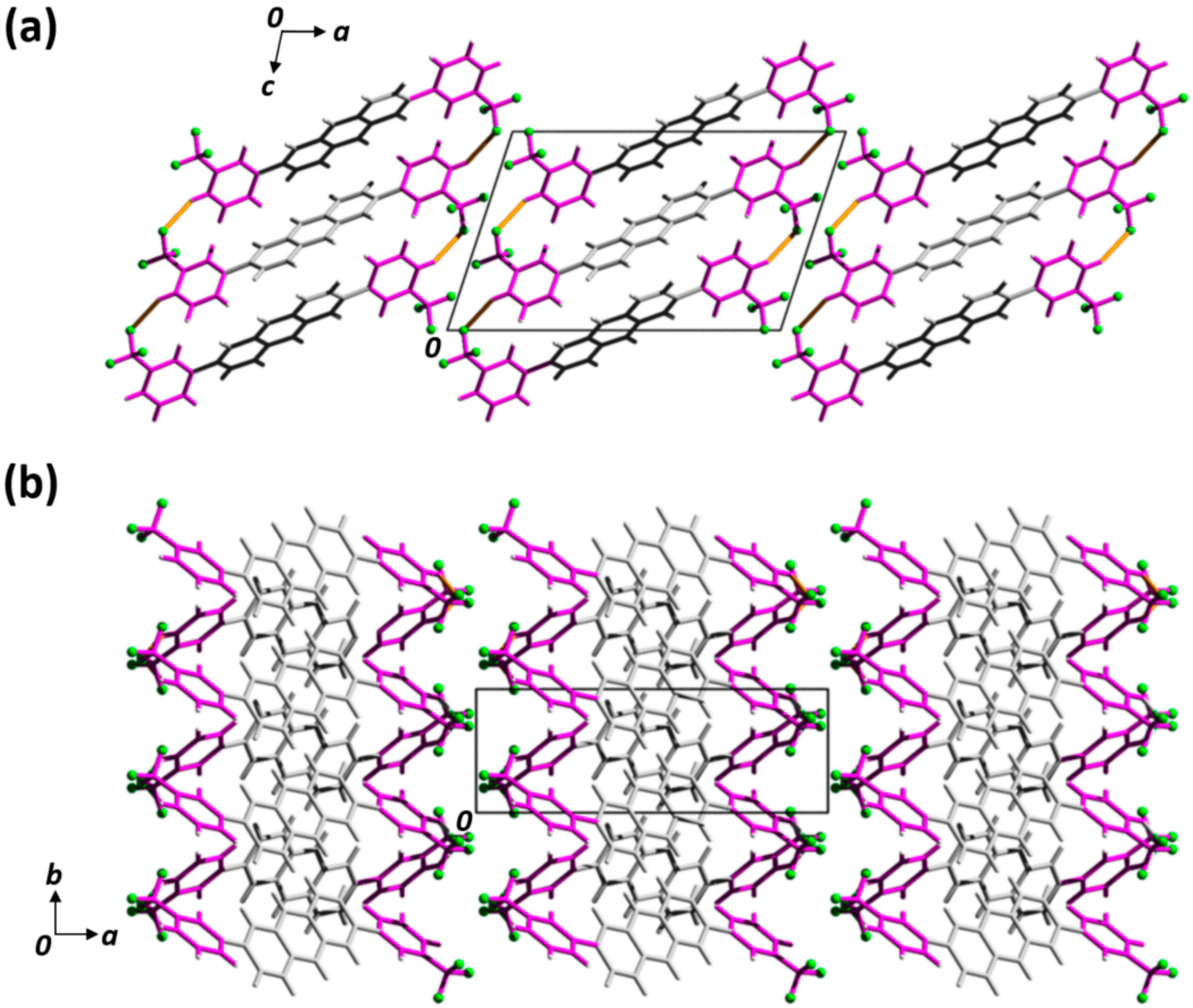

Figure S11. (**a**) Molecular of m-CF_3Ph viewed along the b-direction (**b**) and c-direction. 2,6-position moieties are shown in magenta, and anthracene cores are shown in grey, while fluorine atoms are shown in green. Short contacts between molecules (within array) are shown in yellow.

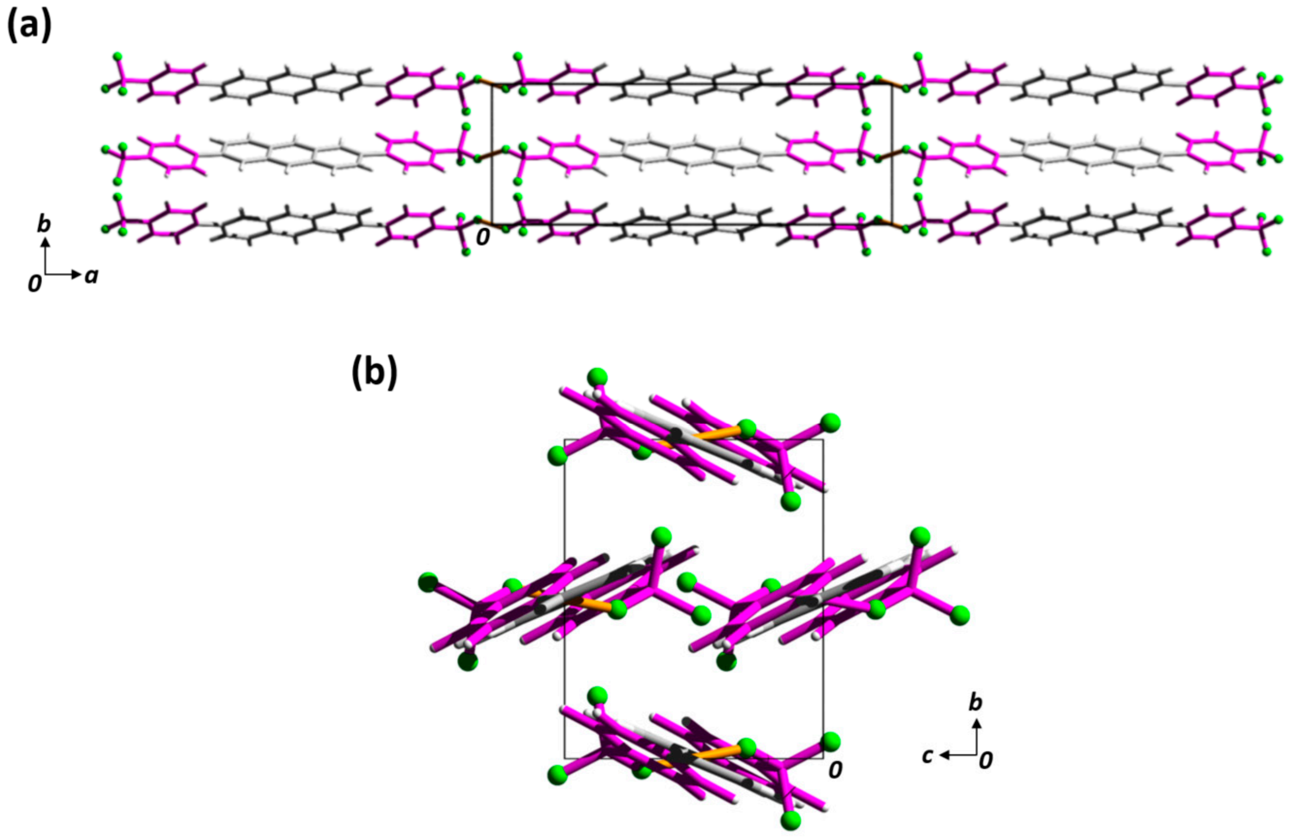

Figure S12. (**a)** Packing of p-CF_3Ph viewed along the *c*-direction (**b**) and *a*-direction. 2,6-position moieties are shown in magenta, and anthracene cores are shown in grey, while fluorine atoms are shown in green. Short contacts between molecules (within array) are shown in yellow.

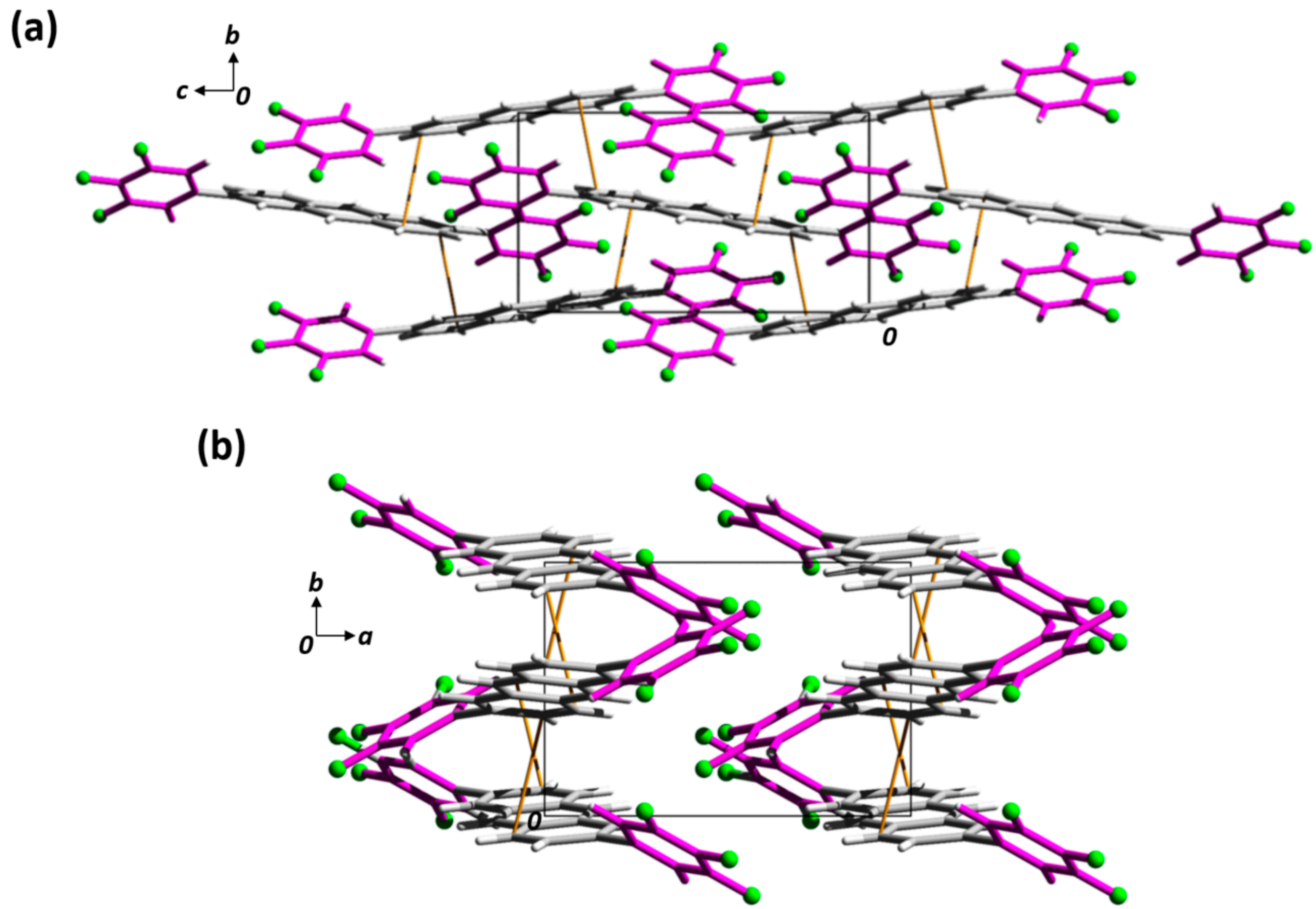

Figure S13. (**a**) Packing of 3,4,5-F_3Ph viewed along the *a*-direction (**b**) and *c*-direction. 2,6-position moieties are shown in magenta, and anthracene cores are shown in grey, while fluorine atoms are shown in green. Short contacts between molecules (within array) are shown in yellow.

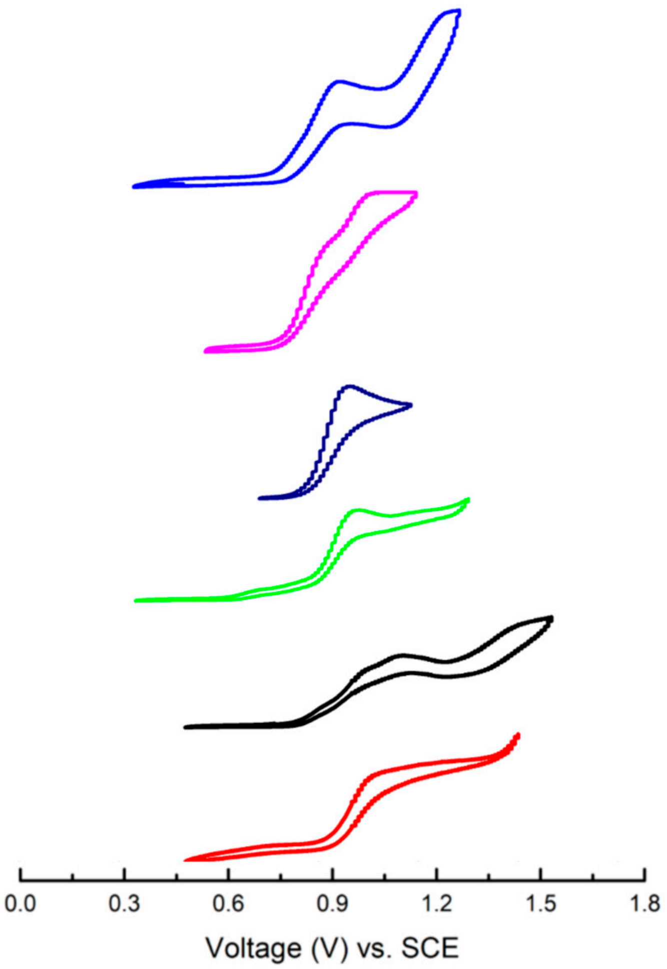

Figure S14. A quasi-reversible oxidation process is observed for compounds with similar halfway oxidation potentials**,** o-FPh (blue), m-FPh (magenta), p-FPh (navy), m-CF_3Ph (green), p-CF_3Ph (black) and 3,4,5-F_3Ph (red). The HOMO energy levels (E_{HOMO}) were estimated using the onset of the oxidation potentials.

© 2020 by the authors. Submitted for possible open access publication under the terms and conditions of the Creative Commons Attribution (CC BY) license (http://creativecommons.org/licenses/by/4.0/).
